# Impact of a sudden drop in ambient temperature on some phenotypic and functional properties of the camel’s immune system

**DOI:** 10.5455/javar.2025.l954

**Published:** 2025-09-22

**Authors:** Fathi Ahmed AL-Musallam, Baraa Falemban, Mayyadah Abdullah Alkuwayti, Najla K. Al Abdulsalam, Hind Althagafi, Jamal Hussen

**Affiliations:** 1 Department of Microbiology, College of Veterinary Medicine, King Faisal University, Al-Ahsa, Saudi Arabia; 2 Department of Biological Sciences, College of Science, King Faisal University, Al Ahsa, Saudi Arabia; 3 Department of Biology, College of Science, Princess Nourah Bint Abdulrahman University, Riyadh, Saudi Arabia

**Keywords:** Camel, cold stress, cortisol, immunophenotype, leukocytes, phagocytosis

## Abstract

**Objective:**

There is a particular lack of studies on the immune response of camels to cold stress conditions. The present study aimed, therefore, at the *ex vivo* investigation of the effect of a sudden decline in ambient temperature on some phenotypic and functional immunological parameters in camels.

**Materials and Methods:**

Using flow cytometry and antibody staining, leukocyte composition, distribution of lymphocyte subsets, and the expression of some cell activation markers were analyzed in camels under normal temperatures and a few days following a sudden environmental temperature decrease. In addition, phagocytosis activity and capacity of neutrophils and monocytes incubated *ex vivo* with Zymosan A Bioparticles or *Staphylococcus* *aureus* were comparatively investigated before and after cold exposure of the camels.

**Results:**

Exposure of the camels to low ambient temperatures resulted in a significant increase in the total white blood cell count and the absolute counts of neutrophils and lymphocytes. On the other hand, the decrease in monocyte counts after cold exposure resulted in a decreased lymphocyte-to-monocyte ratio. In addition, *ex vivo* analysis of phagocytosis and activation marker expression revealed reduced phagocytosis activity and capacity, as well as the downregulation of the activation markers *CD44* and lymphocyte function-associated antigen 1 on leukocytes from the camels after cold exposure.

**Conclusion:**

The current study identified a significant impact of exposure to low ambient temperatures on the distribution of leukocyte subpopulations in camel blood. In addition, *ex vivo* analysis of phagocytosis revealed the impaired innate antimicrobial function of phagocytes in camels under cold stress. The underlying mechanisms for the observed effects of cold stress on the camel’s immune system and their clinical significance for camel health remain to be elucidated in further studies.

## Introduction

The dromedary camel (*Camelus dromedarius*) is well-adapted to the desert with the ability to grow, reproduce, and produce milk under harsh environments. Thanks to their special thermoregulation system, camels display a relatively constant body temperature with minimal daily fluctuations, even during the hot summer [1, 2]. The range of ambient temperature in which camels still display this thermal constant state (known as the thermal neutral zone) has been recently estimated between 10°C and 40°C [3], a range that is expansive when compared to other livestock species [4]. To maintain this thermal state under heat or cold stress conditions, camels employ specialized cooling or heating thermoregulation processes to prevent hyperthermia or hypothermia, respectively [5, 6].

When it comes to scientific research, camels lagged behind other livestock species for a long time. However, recently, camel research has gradually gained increased interest. One of the most extensively investigated characteristics of camels is their physiological adaptation to thermal stress. The majority of studies, however, focused on the adaptation of several body systems in camels to heat stress conditions [7, 8]. On the other hand, as climate change also causes colder winters in the desert environment, camels may experience cold stress conditions during the winter season. Studies in several species have highlighted the impact of cold stress on growth performance, metabolic activity, and the immune response [9–13]. For example, cold stress in pigs was associated with increased levels of serum adrenalin, cortisol, IL-1 beta (IL-1b), and IL-6 with enhanced upregulation of tissue cytokines in response to LPS stimulation [14]. Similarly, in chickens, cold stress enhanced the expression level of the cytokines IL-1b, IL-6, IL-12, and IL-4, indicating a stimulatory effect on innate inflammatory cytokines as well as adaptive type 1 and type 2 immune responses [15]. After 1 week of low-temperature exposure, rats showed an upregulation of genes related to anti-inflammatory mediators, while pro-inflammatory marker genes were downregulated in the white adipose tissue [16]. According to another study in rats, short-term cold stress resulted in decreased plasma levels of the T-cell cytokines IFN-γ, IL-2, and IL-4 and the development of regulatory *CD4*+*CD25*+*Foxp3*+ T cells [13].

Physiological stress leukogram, mainly induced by elevated levels of cortisol and catecholamines, such as epinephrine, is usually a result of changes in the distribution of peripheral leukocyte subsets. By mobilizing the pool of marginal neutrophils into the circulation and extending the half-life of these cells, those mediators induce an increased number of blood neutrophils [17]. This neutrophilia, together with the cortisol-induced lymphopenia as a result of the redistribution of cells to lymphatic tissues, leads most often to a marked increase in the neutrophil-to-lymphocyte ratio (NLR), a sensitive marker for physiological stress [18].

Limited data are available on the impact of cold stress on the camel’s immune system. This study was, therefore, conducted to investigate the impact of a sudden drop in ambient temperature on some parameters of the camel’s immune system.

## Materials and Methods

### Ethical approval

This study was conducted in line with the principles of the Declaration of Helsinki. Approval was granted by the Ethics Committee of King Faisal University, Saudi Arabia (KFU-REC-2024-JUN-ETHICS1843).

### Study location and experimental design

The study was conducted in the eastern province of Saudi Arabia (Al-Ahsa region; N 25°17^�^8.0844^��^, E 49°29^�^11.3316^��^), an arid region with a desert climate characterized by hot (40°C to 50°C day temperatures) and dry summers extending from April to November, while the winter is usually short and moderate (December to March) with occasional showers (day temperatures range between 15°C and 29°C). The study was planned during a sudden decline in ambient temperature between the end of November and the beginning of December 2024. Blood sampling was performed where the animals were still on the farm at 06:00 am on 27.11.2024 (ambient temperature 20°C) before the temperature drop and, on 03.12.2024, 6 days after the exposure to cold temperatures (ambient temperature 12°C).

### Animals

The study was conducted on a dromedary camel herd reared on a private camel farm in the Al-Ahsa region (Eastern Province in Saudi Arabia). Ten camels were selected from the 22 animals on the farm to be included in the study. The study population included five male and five female camels, aged between 1 and 6 years, with a mean age and a SD of 2.7 ± 1.6 years. All the female camels were non-lactating, with one pregnant and 4 non-pregnant she-camels. The camels were kept under a traditional management system, where the camels were grazing during the daytime (06:00 a.m. to 07:00 p.m.) and returned to the farm in the evening to be corralled in a group-field fence during the nighttime (07:00 p.m. to 06:00 a.m.). Being housed in an open-fence barn, the camels were directly exposed to changes in ambient temperatures. For some camels, especially pregnant and young camels, the farmer used textile covers to warm the camels during the night. The camels were fed on hay, barley, bread, and dates. Drinking water was available for the camels in the stall and the grazing range. Ambient temperatures (Table 1) were measured three times daily at 12:00 am, 06:00 am, and 12:00 pm using a digital thermometer (ThermoPro TP50 Digital Hygrometer).

### Blood collection and processing

Blood (5 ml) was collected from the jugular vein into vacutainer tubes containing EDTA (ethylenediaminetetraacetic acid) for cell separation or into tubes without anticoagulants (Guangzhou Improve Medical Instruments Co., Ltd., Guangzhou, China) for serum collection, and the collected samples were transported to the laboratory within two hours. Serum samples were separated after blood centrifugation (1,000 × *g*; 15 min; room temperature, RT) and were stored at −80°C.

**Table 1.** Ambient temperature (°c).

Time	20-Nov	21-Nov	22-Nov	23-Nov	24-Nov	25-Nov	26-Nov	27-Nov	28-Nov	29-Nov	30-Nov	1-Dec	2-Dec	3-Dec
12:00 AM	21	20	20	20	19	20	23	24	20	14	14	12	13	13
6:00 AM	31	31	28	28	29	29	31	20	16	11	12	12	12	12
12:00 PM	34	34	31	30	31	32	34	33	18	19	20	19	20	20
Average	28.7	28.3	26.3	26.0	26.3	27.0	29.3	25.7	18.0	14.7	15.3	14.3	15.0	15.0

### Enzyme-linked immunosorbent assay (ELISA) for cortisol measurement

A commercial ELISA test kit (kit sensitivity of 1.0 ng/ml and kit detection range of 1.3–800 ng/ml) was used for the analysis of serum cortisol based on manufacturer guidelines (DRG, Springfield, NJ 07081 USA). Briefly, each well of an ELISA plate pre-coated with anti-cortisol antibodies received a 20 µl serum sample together with 100 µl of horseradish peroxidase-conjugated cortisol. After incubation for 1 h at RT, the kit washing buffer was used to wash the wells (three times). Subsequently, 100 µl of the ready-to-use substrate and chromogen solution (3,3^�^,5,5^�^-Tetramethylbenzidine) was added to the wells for 20 min in the dark, followed by stopping the reaction using 100 µl of the stop solution (0.16M H₂SO₄). Finally, the optical density values were measured using an ELISA reader (iMark Bio-Rad Laboratories). A standard semi-logarithmic curve fit was prepared using different standard cortisol concentrations and used for the calculation of cortisol concentrations in the samples.

### Cell separation for flow cytometry

Cell separation was performed as previously described [7]. Briefly, red blood cells were lysed by incubating a 1 ml blood sample with 5 ml distilled water for 20 sec in a 15 ml tube, followed by the addition of 5 ml 2× PBS and centrifugation at 3,000 RPM for 10 min at 10°C. This lysis step was repeated twice with centrifugation at 2,200 and 1,500 RPM for 10 min. Finally, the pellet was resuspended in cold PBS and adjusted to 1 × 10^6^ cells/ml. Cell vitality was measured after the addition of 7-AAD (7-amino-actinomycin D) and was always above 90%.

### Flow cytometric analysis of leukocyte subsets

Separated leukocytes were labeled with monoclonal antibodies (mAbs) to the leukocyte antigens *CD44*, leukocyte function antigen 1 (LFA1; known as *CD11a*), major histocompatibility complex (MHC) class-II molecules, BAQ44A (B2 cell), *WC1* (gd T cell), *CD4* (helper T cell), *CD172a*, *CD14*, and *CD163* [7, 19]. Cell staining was performed in two steps, where cells were first incubated in a 96-well plate (1 × 10^6^ cells/well) with the primary mAbs for 15 min at 4°C. After washing with PBS/BSA buffer, the second staining step was done by incubating the cells with fluorochrome-labeled antibodies to mouse IgM, IgG1, and IgG2a (Invitrogen). Finally, the cells were washed and analyzed by flow cytometry (Becton Dickinson Accuri C6 flow cytometer; Becton Dickinson Biosciences, San Jose, California, USA).

### Analysis of phagocytosis

*In vitro* phagocytosis of Zymosan A Bioparticles and *Staphylococcus aureus* by neutrophils and monocytes was analyzed by flow cytometry as described previously [7]. Leukocytes were plated in a 96-well plate (1 × 10^5^ cells/well) in 100 µl RPMI medium. The cells were then incubated with 100 µl/well of pHrodo™ Green Zymosan A Bioparticles^®^ Conjugate (Invitrogen, Germany) resuspended in RPMI medium at 0.05 mg/ml and homogenized by vortexing or 50 µl FITC-conjugated *S*. *aureus* (2 × 10^8^ cells/ml in RPMI medium; Institute of Immunology, Hannover University of Veterinary Medicine, Germany). After incubation for 45 min at 37°C, 7-AAD (100 µl/well) was added to the cells, and the analysis was performed on the Accuri flow cytometer.

### Statistical analyses

The column statistic function was used to calculate means and SEM using the Prism software (GraphPad). The Shapiro-Wilk test was used for data normality testing. The comparison between means was performed using a paired Student’s *t*-test or Mann-Whitney test (for not normally distributed values)*, *with* p-*values less than 0.05 indicating significant effects.

## Results

### Cortisol levels in camel serum

The analysis of cortisol levels in camel serum revealed a mean cortisol concentration of 10.1 ± 1.6 ng/µl with values ranging between 5 and 19 ng/µl (min. and max.). Camels displayed significantly higher concentrations of cortisol (15.6 ± 2.8 ng/µl) in their serum after exposure to low temperatures (*p* < 0.05).

### Camel leukogram pattern associated with cold stress

**Figure 1.**
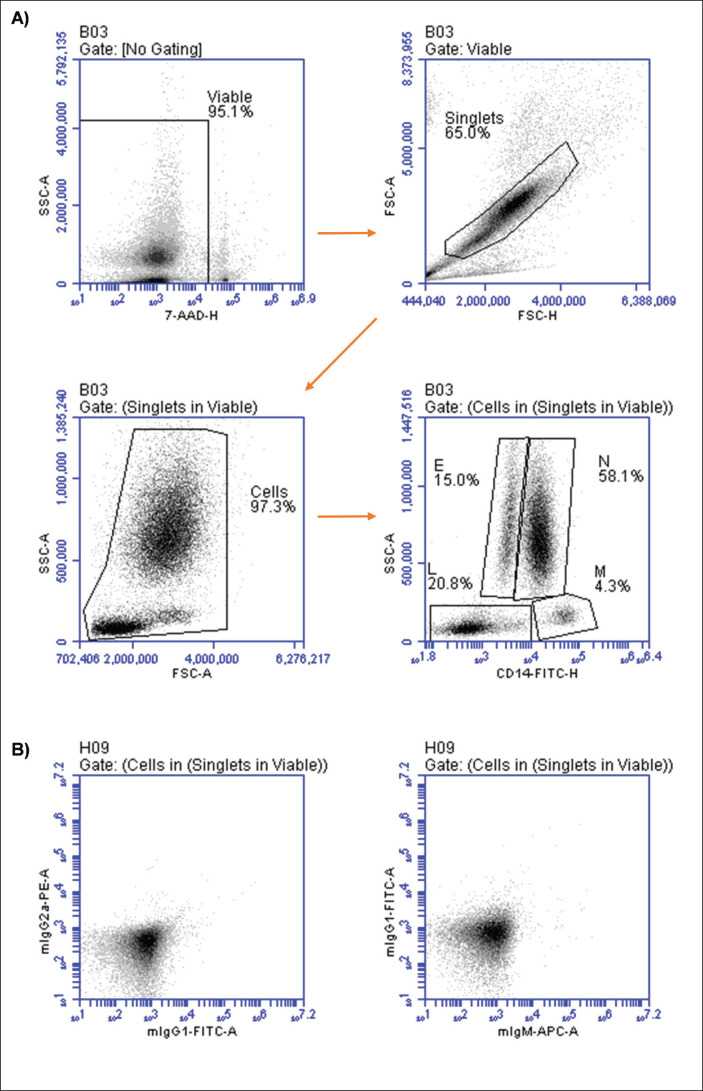
Gating strategy for flow cytometric analysis of leukocyte populations in camel blood. Leukocytes were labeled with antibodies to camel *CD14* (A) or isotype control antibodies (B) and analyzed by flow cytometry. After gating on live cells (cells negative for 7-AAD), singlets were gated based on FSC-H and FSC-A properties. Gates were set on neutrophils (N), eosinophils (E), monocytes (M), and lymphocytes (L); their percentages were calculated based on SSC-A and staining with *CD14* antibodies.

Two days after the collection of the first blood sample, one camel showed signs of weakness, limited movement, and reduced appetite, and was therefore excluded from the analysis. As shown in Figure 1, the relative distribution of camel leukocyte populations in blood was analyzed by flow cytometry. To do so, camel neutrophils, eosinophils, monocytes, and lymphocytes were identified based on their side scatter values and staining patterns with *CD14* antibodies after the exclusion of dead cells and cell doublets (Fig. 1A). Figure 1B shows control staining, with no cells stained positively with isotype control antibodies.

**Figure 2.**
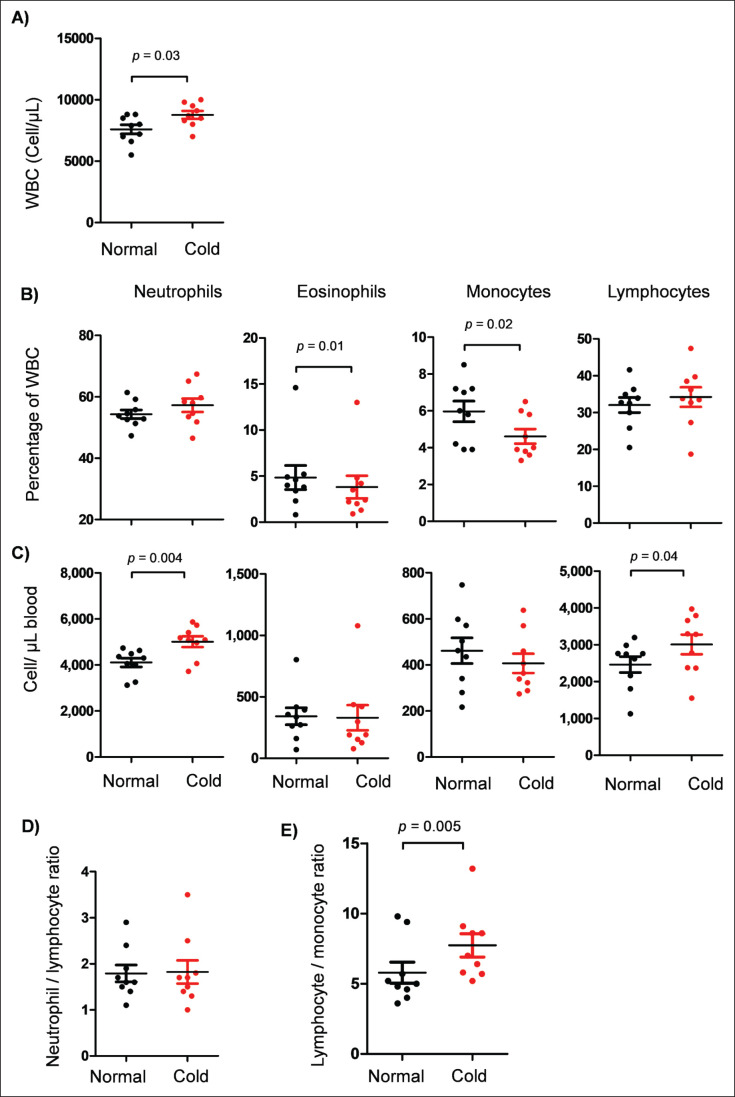
Leukocyte populations in camel blood. A) The total number of WBC was counted using the Neubauer cell counter and light microscopy. B) Relative distribution of camel leukocyte populations in blood was analyzed by flow cytometry. C) Absolute numbers of leukocyte populations were calculated by multiplying their relative percentages of leukocytes by the total leukocyte count.

The total white blood cell (WBC) counts were counted using a Neubauer cell counter and light microscopy after diluting blood samples with Turk solution (1:10) to lyse red blood cells and stain WBCs. The relative and absolute counting of blood leukocytes and their subpopulations are presented in Figure 2. The absolute numbers of leukocyte subpopulations were calculated by multiplying their relative percentages (determined by flow cytometry) by the total WBC count. As shown in Figure 2A, the total WBC count was significantly increased after cold exposure (8,767 ± 317 cells/µl blood) compared to the WBC count under normal temperatures (7,589 ± 370 cells/µl blood). After cold exposure, the camels showed significantly reduced percentages of eosinophils (3.8% ± 1.2% of WBC) and monocytes (4.6% ± 0.3% of WBC) compared to the percentage of eosinophils (4.8% ± 1.3% of WBC) and monocytes (5.9% ± 0.5% of WBC) under normal temperatures (*p *< 0.05) (Fig. 2B). The absolute numbers of leukocyte populations (Fig. 2C) revealed significantly (*p* < 0.05) increased numbers of neutrophils (5,009 ± 235 versus 4105 ± 191 cells/µl blood under normal temperatures) and lymphocytes (3,008 ± 266 versus 2461 ± 217 cells/µl blood under normal temperatures). While the NLR was not affected by cold exposure for the camels (*p* > 0.05) (Fig. 2D), a significant increase was observed in the lymphocyte-to-monocyte ratio (LMR) for camels under cold temperatures compared to normal temperatures (*p* < 0.05) (Fig. 2E).

### Impact of cold exposure on the distribution of lymphocyte subsets in camel blood

**Figure 3.**
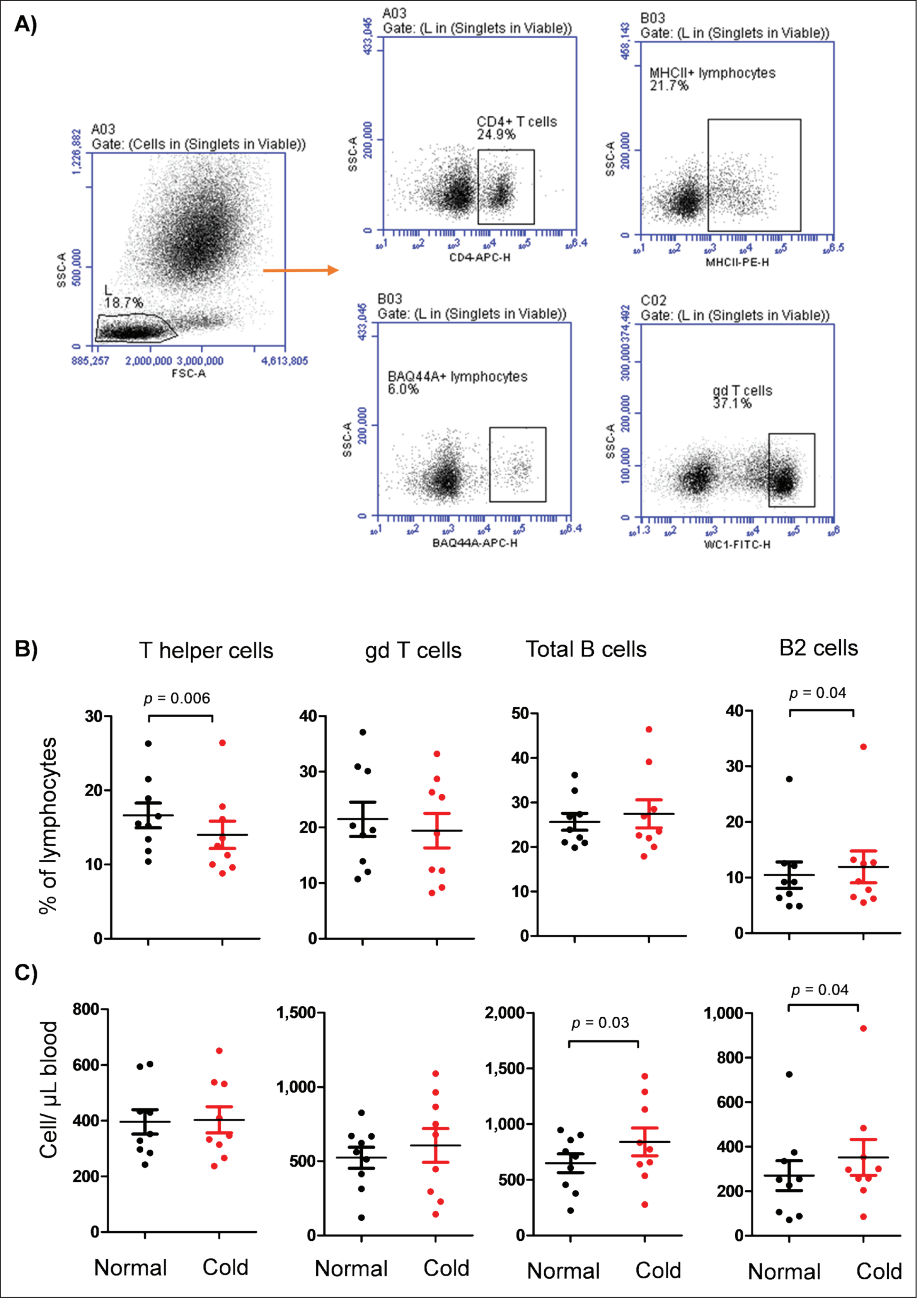
Flow cytometric analysis of lymphocyte subsets in blood. A) gating strategy for the analysis of lymphocyte subsets in blood. After gating lymphocytes based on FSC and SSC parameters, *CD4 *+ cells (T helper cells), *MHC II*+ cells (B cells), BAQ44A+ cells (B2 cells), and *WC1*+ cells (gd T cells) were identified based on their positive reactivity with marker antibodies. B) The percentage of T helper cells, total B cells, B2 cells, and gd T cells was calculated and presented as dot plots.

Camel lymphocyte subsets were identified using immune staining and flow cytometry. As shown in Figure 3A, the percentages of *CD4*+ helper T cells, total B cells, B2 cells, and gd T cells were calculated based on the positive staining of lymphocytes with monoclonal antibodies to *CD4*, *MHC II*, BAQ44A, and *WC1* molecules, respectively. Lymphocyte composition after cold exposure was characterized by a significant expansion in the fraction of B2 cells (BAQ44A+ lymphocytes), while the percentage of helper T cells was significantly reduced when compared to their values under normal temperatures (*p* < 0.05) (Fig. 3B). Absolute counting of lymphocyte subsets revealed higher numbers of total B cells (*MHC* *II*+ lymphocytes) as well as B2 cells in the camels after cold exposure than under normal temperatures (*p* < 0.05) (Fig. 3C).

### Impact of cold stress on activation marker expression on camel leukocyte populations

**Figure 4.**
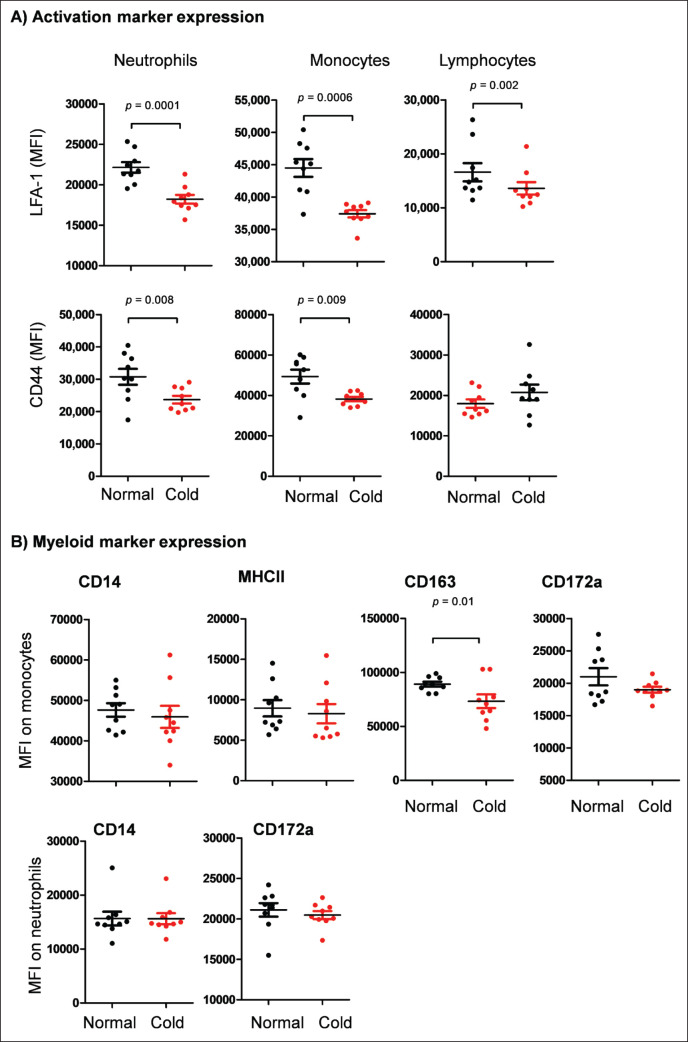
A) Expression level of the cell activation molecules, leukocyte function antigen 1 (*LFA-A*; *CD11a*) and *CD44*. Leukocytes were labeled with monoclonal antibodies to *CD11a* and *CD44* and analyzed by flow cytometry. The expression levels of the surface markers LFA-1 and *CD44* were calculated as mean fluorescence intensity (MFI) for neutrophils, monocytes, and lymphocytes. B) Impact of cold exposure on the expression of selected myeloid markers on neutrophils and monocytes. Leukocytes were labeled with antibodies to *CD14*. *MHC II*, *CD163*, and *CD172a,* and labeled cells were analyzed by flow cytometry. The expression level of the cell markers was presented as the mean fluorescence intensity of the selected marker on monocytes and neutrophils.

The abundance of the cell activation markers LFA1 and *CD44* was analyzed by flow cytometry by calculating the mean fluorescence intensity (MFI) of cell populations stained positively with antibodies to *CD11a* and *CD44*. For blood leukocytes separated from camels after exposure to cold stress, a significant decrease (*p* < 0.05) in the abundance of the cell adhesion molecule lymphocyte function-associated antigen 1 (LFA-1) was observed on neutrophils, monocytes, and lymphocytes (Fig. 4A) when compared to cells separated from the same camels under normal temperatures. Similarly, the expression level of *CD44* was significantly reduced on the surface of neutrophils and monocytes after camel exposure to cold stress (Fig. 4A). No significant change was, however, observed in the abundance of *CD44* in lymphocytes after cold exposure to camels (*p* > 0.05) (Fig. 4A).

### Impact of cold exposure on the expression of some cell markers on neutrophils and monocytes

The expression density of the cell markers *CD163*, *CD14*, *CD172a*, and *MHC II* on camel leukocytes was analyzed by flow cytometry and presented comparatively as MFI values for monocytes and neutrophils from camels before and after cold exposure (Fig. 4B). Exposure to cold stress resulted in a significant (*p* < 0.05) decrease in the expression density of the surface marker *CD163* (Fig. 4B) on monocytes (73,252 ± 6,368 MFI) in comparison to the density on monocytes from camels under normal temperatures (89,174 ± 2,229 MFI). No significant effect (*p* > 0.05) of cold stress was observed on the expression density of *MHC II* on monocytes or *CD14* and *CD172a* on monocytes and neutrophils (Fig. 4B).

### Impact of cold exposure on phagocytosis function of neutrophils and monocytes

**Figure 5.**
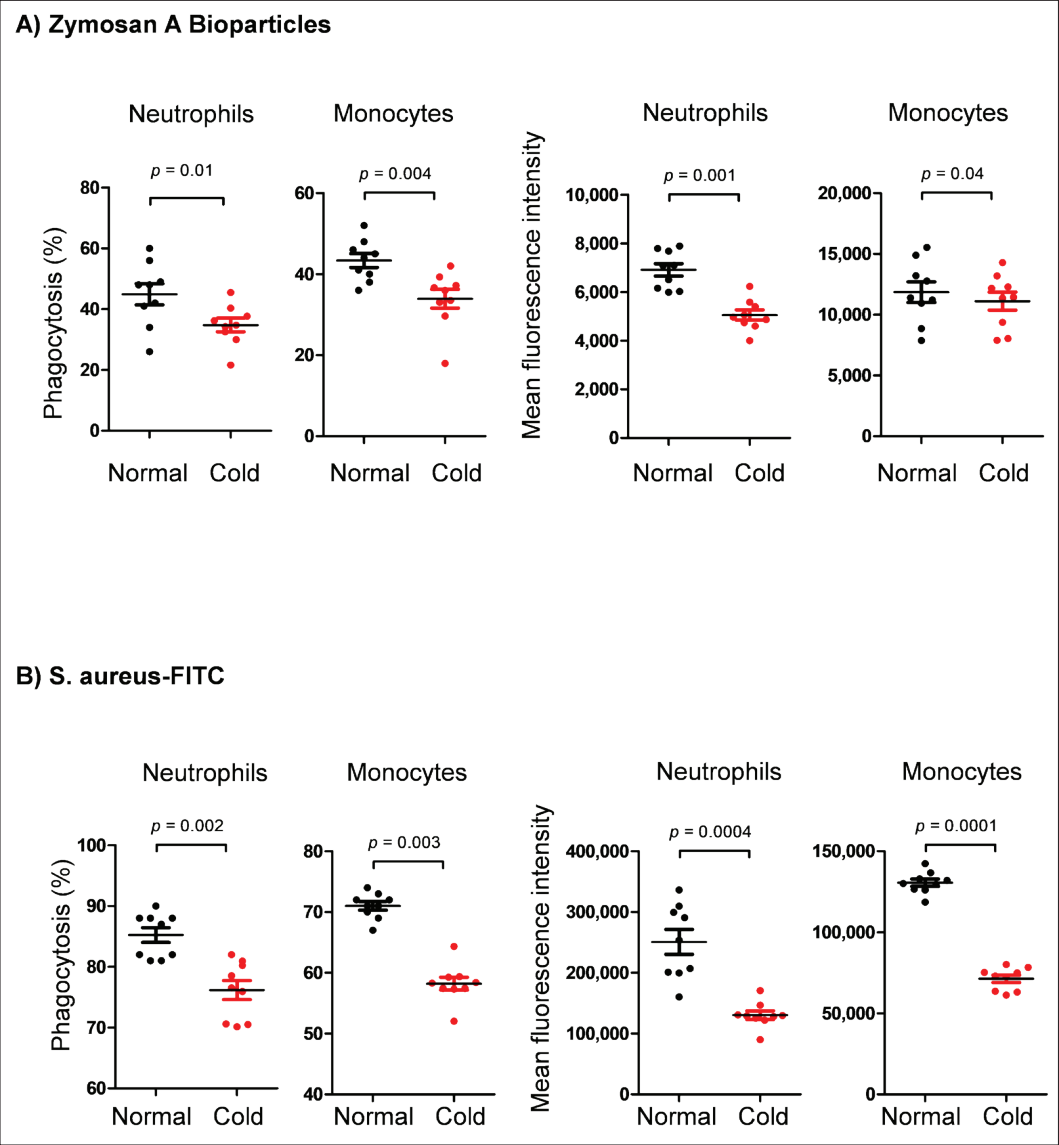
Phagocytosis analysis. Leukocytes were incubated with (A) pHrodo^™^ Green Zymosan A Bioparticles^®^ Conjugate or (B) FITC-conjugated *S*. *aureus* and analyzed by flow cytometry. The percentage of phagocytic neutrophils and monocytes was calculated based on the fraction of cells with increased fluorescence in the FL1 channel. In addition, phagocytosis capacity (number of particles ingested by each phagocyte) was calculated as mean fluorescence intensity (MFI).

The innate function of neutrophils and monocytes was evaluated by the analysis of their capacity to phagocytose Zymosan A Bioparticles and *S*. *aureus* bacteria using flow cytometry. The percentage of phagocytosis-positive cells, as well as their phagocytosis capacity (as an indicator for the number of particles ingested by each phagocyte), was calculated and presented for the camels before and after cold exposure (Figs. 5A, 5B). For the Zymosan A Bioparticles, the mean percentage of phagocytic cells was 44.9% ± 3.5% of total neutrophils and 43.3% ± 1.7% of total monocytes in camels under normal temperatures. After exposure to cold temperatures, the percentage of phagocytic cells was significantly reduced to 34.7% ± 2.2% of total neutrophils and 33.9% ± 2.3% of total monocytes (*p* < 0.05) (Fig. 5A). Similarly, the mean fluorescent intensity of phagocytosis-positive cells was significantly lower for both neutrophils and monocytes from animals under cold stress (Fig. 5A). Phagocytosis of *S*. *aureus* by neutrophils and monocytes showed similar dynamics with significantly lower activity and capacity for cells from animals under cold temperatures than under normal temperatures (*p* < 0.05) (Fig. 5B).

## Discussion

Thermoregulation in camels and the capacity of this species to adapt to high-temperature variation have been investigated in several studies [20]. The majority of studies, however, focused on the adaptation of different body systems in camels to heat stress conditions [7, 21]. On the other hand, studies on the impact of cold stress on camel physiology are scarce. There is a particular lack of studies on the immune response of camels to cold stress conditions. The present study aimed, therefore, to investigate the *ex vivo* effect of a sudden ambient temperature drop on certain phenotypic and functional immunological parameters in camels. To achieve this, leukocyte composition, distribution of lymphocyte subsets, the expression of some activation markers, and the phagocytosis activity and capacity of neutrophils and monocytes were analyzed in camels under normal temperatures, and in the same camels, a few days following sudden environmental temperature decreases.

The changes in camel leukogram observed in this study, with increased total WBC numbers and increased numbers of neutrophils and lymphocytes but a lower percentage of monocytes in camels under cold exposure, seem in agreement with a previous study that reported similar seasonal leukocyte dynamics in camels during winter months compared to other seasons [22]. The cold-stress-induced decrease in monocytes is also supported by a previous controlled study in rats showing a progressive decrease in TLR4+ mononuclear cells in the spleen, mainly resembling monocytes and macrophages, along with decreased temperatures [13]. Together, the observed decrease in monocytes and the reduced expression of the hemoglobin receptor *CD163* seem in agreement with the well-documented effect of stress hormones on blood monocytes [3]. Although the observed increase in neutrophils, the decrease in monocytes, and the elevated LMR are all indicators of a stress leukogram pattern, the lack of lymphopenia with no change in the neutrophils-to-lymphocytes ratio, two important physiological stress markers, argues against this pattern. The question of whether this finding may be associated with the stability of *CD44* expression on lymphocytes, in contrast to other leukocyte populations, or whether exposure of camels to lower ambient temperatures or prolonged periods would elicit modifications in lymphocyte characteristics, remains to be elucidated in forthcoming research.

Studies in rodents showed that housing at sub-optimal temperatures is associated with reduced energy availability for driving the immune responses, resulting in significant changes in the functionality of the immune system [12]. The phagocytes, neutrophils, and monocytes represent innate cells of the first line of defense and are recruited immediately after the onset of infection to phagocytose and kill pathogens [23]. To see whether the observed change in immune cell composition is also associated with changes in their functional activities, microbial phagocytosis by blood phagocytes was investigated *ex vivo*. The marked decrease in the percentage of phagocytic neutrophils and monocytes, as well as the phagocytosis capacity, for both the Zymosan A Bioparticles and the *S*. *aureus* bacteria, reveals a negative effect of cold stress on phagocytosis function in camels. Although empirical evidence from *in vivo* studies involving the experimental infection of camels with microbial pathogens remains unsubstantiated, the observed reduction in phagocytic activity indicates a detrimental influence of cold stress on the innate immune system of camels, resulting in compromised pathogen clearance and an increased sensitivity to infectious diseases. This conclusion, however, needs confirmation through the analysis of additional antimicrobial functions such as the production of reactive oxygen species, NET formation, and degranulation under cold stress conditions. Furthermore, elucidating the mechanistic implications of impaired phagocytic capability necessitates additional investigations into the modulatory influence of cold stress on the expression of phagocytosis receptors, such as IgG receptors, complement receptors, scavenger receptors, and toll-like receptors on camel phagocytes. Research conducted on rodent models indicates that housing at sub-optimal temperatures correlates with diminished energy availability essential for driving immune responses, consequently leading to notable alterations in immune functionality [12]. Hence, constrained energy availability may have contributed to the diminished phagocytic activity observed in camels subjected to cold stress in the present study.

As a fully functional phagocytic receptor with the capacity to initiate the ingestion of large particles [24], the precise role of the hyaluronic acid receptor *CD44* in the compromised phagocytic activity of neutrophils and monocytes under cold stress warrants further investigation in forthcoming studies. Such investigations may encompass the utilization of *CD44*-blocking antibodies to assess their influence on microbial adhesion and/or uptake. Likewise, although the diminished abundance of the LFA-1 [25] across all leukocyte subsets indicates an impaired immune response in camels subjected to cold stress, exploring the clinical significance of the downregulation of this cell adhesion molecule and understanding their role in the observed change in phagocytosis activity upon cold exposure remain interesting areas of research.

As no impact of cold exposure was found on the numbers of helper T cells or gd T cells, the observed increase in lymphocytes seems to be due to the selective increase in peripheral B lymphocytes. One possible explanation for this could be the stress-induced increase in intestinal barrier permeability [26], leading to the translocation of the bacterial endotoxin LPS [27], which is known for its mitogenic activity on B cells [28, 29]. An argument against this assumption is, however, the phenotype of monocytes with no change in *MHC II* expression, a marker of LPS stimulation in camels [30].

The findings of the present investigation were derived from specimens obtained from a mere nine camels (after the exclusion of one diseased animal), which constitutes a limitation of the present study. Furthermore, the outcomes of the current study may have been influenced by the variability within the animals due to the implementation of textile coverings on certain camels. This highlights the need for future studies using a higher number of camels with minimal inter-animal variability to mitigate any potential impact on the statistical robustness of the findings.

## Conclusion

The current study identified a significant impact of exposure to low temperatures on the distribution of leukocyte subpopulations in camel blood. The observed cold-associated leukogram pattern suggests a complex interplay between cold exposure and the immune system, highlighting the need for further studies to uncover the underlying mechanisms. In addition, *ex vivo* analysis of phagocytosis revealed the impaired innate antimicrobial function of phagocytes in camels under cold stress.

## List of abbreviations

CD, Cluster of differentiation; EAE, Experimental autoimmune encephalomyelitis; EDTA, Ethylenediaminetetraacetic acid; ELISA, Enzyme-linked immunosorbent assay; FITC, Fluorescein isothiocyanate; FSC, Forward scatter; IFN, Interferon; IL, Interleukin; LFA, Lymphocyte function-associated antigen; LPS, Lipopolysaccharide; MFI, Mean fluorescence intensity; NLR, Neutrophil-to-lymphocyte ratio; PBS, Phosphate-buffered saline; RPMI, Roswell Park Memorial Institute (medium); WBC, White blood cells; h, hour; mg, milligram; ml, milliliter; ng, nanogram.
